# Primary decomposition of the ideal of polynomials whose fixed divisor is divisible by a prime power^☆^

**DOI:** 10.1016/j.jalgebra.2013.09.016

**Published:** 2014-01-15

**Authors:** Giulio Peruginelli

**Affiliations:** Institut für Analysis und Comput. Number Theory, Technische Universität, Steyrergasse 30, A-8010 Graz, Austria

**Keywords:** 13B25, 13F20, Integer-valued polynomial, Image of a polynomial, Fixed divisor, Factorization of integer-valued polynomials, Primary components, Primary decomposition

## Abstract

We characterize the fixed divisor of a polynomial f(X) in Z[X] by looking at the contraction of the powers of the maximal ideals of the overring Int(Z) containing f(X). Given a prime *p* and a positive integer *n*, we also obtain a complete description of the ideal of polynomials in Z[X] whose fixed divisor is divisible by $p^{n}$ in terms of its primary components.

*To Sergio Paolini, whose teachings and memory I deeply preserve*.

## 1 Introduction

In this work we investigate the image set of integer-valued polynomials over Z. The set of these polynomials is a ring usually denoted by:

Int(Z)≑{f∈Q[X]|f(Z)⊂Z}.

Since an integer-valued polynomial f(X) maps the integers in a subset of the integers, it is natural to consider the subset of the integers formed by the values of f(X) over the integers and the ideal generated by this subset. This ideal is usually called the fixed divisor of f(X). Here is the classical definition.

Definition 1.1 Let f∈Int(Z). The *fixed divisor* of f(X) is the ideal of Z generated by the values of f(n), as *n* ranges in Z:

d(f)=d(f,Z)=(f(n)|n∈Z).

We say that a polynomial f∈Int(Z) is *image primitive* if d(f)=Z.

It is well-known that for every integer n⩾1 we have

d(X(X−1)⋯(X−(n−1)))=n!

so that the so-called binomial polynomials $B_{n}(X)≑X(X-1)\cdots (X-(n-1))/n!$ are integer-valued (indeed, they form a free basis of Int(Z) as a Z-module; see [4]).

Notice that, given two integer-valued polynomials *f* and *g*, we have d(fg)⊂d(f)d(g) and we may not have an equality. For instance, consider f(X)=X and g(X)=X−1; then we have d(f)=d(g)=Z and d(fg)=2Z. If f∈Int(Z) and n∈Z, then directly from the definition we have d(nf)=nd(f). If cont(F) denotes the content of a polynomial F∈Z[X], that is, the greatest common divisor of the coefficients of *F*, we have F(X)=cont(F)G(X), where G∈Z[X] is a primitive polynomial (that is, cont(G)=1). We have the relation:

d(F)=cont(F)d(G).

In particular, the fixed divisor is contained in the ideal generated by the content. Hence, given a polynomial with integer coefficients, we can assume it to be primitive. In the same way, if we have an integer-valued polynomial f(X)=F(X)/N, with f∈Z[X] and N∈N, we can assume that (cont(F),N)=1 and F(X) to be primitive.

The next lemma gives a well-known characterization of a generator of the above ideal (see [1, Lemma 2.7]).

Lemma 1.1 *Let*f∈Int(Z)*be of degree d and set*

1) $d_{1}=\sup\{n\in Z|\frac{f(X)}{n}\in \mathrm{Int}(Z)\}$*,*
2) $d_{2}=\mathrm{GCD}\{f(n)|n\in Z\}$*,*
3) $d_{3}=\mathrm{GCD}\{f(0),\ldots ,f(d)\}$*,*

*then*$d_{1}=d_{2}=d_{3}$*.*

Let f∈Int(Z). We remark that the value $d_{1}$ of Lemma 1.1 is plainly equal to:

$$d_{1}=\sup\left\{n\in Z|f\in n\mathrm{Int}(Z)\right\}.$$

Moreover, given an integer *n*, we have this equivalence that we will use throughout the paper, a sort of ideal-theoretic characterization of the arithmetical property that all the values attained by f(X) are divisible by *n*:

f(Z)⊂nZ⟺f∈nInt(Z)

(nInt(Z) is the principal ideal of Int(Z) generated by *n*). From 1) of Lemma 1.1 we see immediately that if f(X)=F(X)/N is an integer-valued polynomial, where F∈Z[X] and N∈N coprime with the content of F(X), then d(f)=d(F)/N, so we can just focus our attention on the fixed divisor of a primitive polynomial in Z[X].

We want to give another interpretation of the fixed divisor of a polynomial f∈Z[X] by considering the maximal ideals of Int(Z) containing f(X) and looking at their contraction to Z[X]. We recall first the definition of unitary ideal given in [12].

Definition 1.2 An ideal I⊆Int(Z) is *unitary* if I∩Z≠0.

That is, an ideal *I* of Int(Z) is unitary if it contains a non-zero integer, or, equivalently, IQ[X]=Q[X] (where IQ[X] denotes the extension ideal in Q[X]). The whole ring Int(Z) is clearly a principal unitary ideal generated by 1.

The next results are probably well-known, but for the ease of the reader we report them. The first lemma says that a principal unitary ideal *I* is generated by a non-zero integer, which generates the contraction of *I* to Z. In particular, this lemma establishes a bijective correspondence between the nonzero ideals of Z and the set of principal unitary ideals of Int(Z).

Lemma 1.2 *Let* I⊆Int(Z) *be a principal unitary ideal. If* I∩Z=nZ *with* n≠0 *then* I=nInt(Z) *. In particular,* nInt(Z)∩Z=nZ *. Moreover,* $n_{1}\mathrm{Int}(Z)=n_{2}\mathrm{Int}(Z)$ *with* $n_{1},n_{2}\in Z$ *if and only if* $n_{1}=\pm n_{2}$ *.*

Proof If I=(f) for some f∈Int(Z) then deg(f)=0 since a non-zero integer *n* is in *I*. Since f(X) is integer-valued it must be equal to an integer and so it is contained in I∩Z=nZ. Hence we get the first statement of the lemma. If $n_{1}\mathrm{Int}(Z)=n_{2}\mathrm{Int}(Z)$ then $n_{1}=n_{2}f$ with f∈Int(Z); this forces *f* to be a non-zero integer, so that $n_{1}$ divides $n_{2}$. Similarly, we get that $n_{2}$ divides $n_{1}$. □

Lemma 1.3 *Let* $I_{1},I_{2}\subseteq \mathrm{Int}(Z)$ *be principal unitary ideals. Then* $I_{1}\cap I_{2}$ *is a principal unitary ideal too.*

Proof Suppose $I_{i}=n_{i}\mathrm{Int}(Z)$, where $n_{i}\in Z$, $n_{i}Z=I_{i}\cap Z$, for i=1,2. We have $n_{1}Z\cap n_{2}Z=nZ$, where $n=\mathrm{lcm}\{n_{1},n_{2}\}$. The ideal $I_{1}\cap I_{2}$ is unitary since $n\in I_{1}\cap I_{2}$. In particular, we have $I_{1}\cap I_{2}⊇n\mathrm{Int}(Z)$. We have to prove that $I_{1}\cap I_{2}\subseteq n\mathrm{Int}(Z)$. Let $f\in I_{1}\cap I_{2}$. Then $f(Z)\subset n_{1}Z\cap n_{2}Z=nZ$, so that $\frac{f(X)}{n}\in \mathrm{Int}(Z)$. □

The previous lemma implies the following decomposition for a principal unitary ideal generated by an integer *n*, with prime factorization $n=\prod_{i}p_{i}^{a_{i}}$. We have

$$n\mathrm{Int}(Z)=\underset{i}{⋂}p_{i}^{a_{i}}\mathrm{Int}(Z)=\prod_{i}p_{i}^{a_{i}}\mathrm{Int}(Z)$$

where the last equality holds because the ideals $p_{i}^{a_{i}}Z$ are coprime in Z, hence they are coprime in Int(Z).

We are now ready to give the following definition.

Definition 1.3 Let f∈Int(Z). The *extended fixed divisor* of f(X) is the minimal ideal of the set {nInt(Z)|n∈Z,f∈nInt(Z)}. We denote this ideal by D(f).

Equivalently, in the above definition, we require that nInt(Z) contains the principal ideal in Int(Z) generated by the polynomial f(X). Lemma 1.2, Lemma 1.3 show that the minimal ideal in the above definition does exist: it is equal to the intersection of all the principal unitary ideals containing f(X). Notice that the extended fixed divisor is an ideal of Int(Z), while the fixed divisor is an ideal of Z. The polynomial f(X) is image primitive if and only if its extended fixed divisor is the whole ring Int(Z). In the next sections we will study the extended fixed divisor by considering the *p*-part of it, namely the principal unitary ideals of the form $p^{n}\mathrm{Int}(Z)$, p∈Z being prime and *n* a positive integer.

The following proposition gives a link between the fixed divisor and the extended fixed divisor: the latter is the extension of the former and conversely. So each of them gives information about the other one.

Proposition 1.1 *Let*f∈Int(Z)*. Then we have*:

a) D(f)∩Z=d(f)*,*
b) d(f)Int(Z)=D(f)*.*

Proof Let d,D∈Z be such that d(f)=dZ and D(f)=DInt(Z). Since d(f)Int(Z)=dInt(Z) is a principal unitary ideal containing f(X), from the definition of extended fixed divisor, we have D(f)⊆dInt(Z). In particular, D⩾d. We also have f(X)/D∈Int(Z) and so d⩾D, by characterization 1) of Lemma 1.1. Hence we get a). From that we deduce that d(f)⊆D(f), so statement b) follows. □

As already remarked in [5], the rings Z and Int(Z) share the same units, namely {±1}. Then [5, Proposition 2.1] can be restated as follows.

Proposition 1.2 **Cahen–Chabert**

*Let* f∈Int(Z) *be irreducible in* Q[X] *. Then* f(X) *is irreducible in* Int(Z) *if and only if* f(X) *is not contained in any proper principal unitary ideal of* Int(Z) *.*

The next lemma has been given in [6] and is analogous to the Gauss Lemma for polynomials in Z[X] which are irreducible in Int(Z).

Lemma 1.4 **Chapman–McClain**

*Let*f∈Z[X]*be a primitive polynomial. Then*f(X)*is irreducible in*Int(Z)*if and only if it is irreducible in*Z[X]*and image primitive.*

For example, the polynomial $f(X)=X^{2}+X+2$ is irreducible in Q[X] and also in Z[X] since it is primitive (because of Gauss Lemma). But it is reducible in Int(Z) since its extended fixed divisor is not trivial, namely it is the ideal 2Int(Z). So in Int(Z) we have the following factorization:

$$f(X)=2\cdot \frac{X^{2}+X+2}{2}$$

and indeed this is a factorization into irreducibles in Int(Z), since the latter polynomial is image primitive and irreducible in Q[X], and by [5, Lemma 1.1], the irreducible elements in Z remain irreducible in Int(Z). So the study of the extended fixed divisor of the elements in Int(Z) is a first step toward studying the factorization of the elements in this ring (which is not a unique factorization domain).

Here is an overview of the content of the paper. At the beginning of the next section we recall the structure of the prime spectrum of Int(Z). Then, for a fixed prime *p*, we describe the contractions to Z[X] of the maximal unitary ideals of Int(Z) containing *p* (Lemma 2.1). In Theorem 2.1 we describe the ideal $I_{p}$ of Z[X] of those polynomials whose fixed divisor is divisible by *p*, namely the contraction to Z[X] of the principal unitary ideal pInt(Z), which is the ideal of integer-valued polynomials whose extended fixed divisor is contained in pInt(Z). It turns out that $I_{p}$ is the intersection of the aforementioned contractions. In the third section we generalize the result of the second section to prime powers, by means of a structure theorem of Loper regarding unitary ideals of Int(Z). We consider the contractions to Z[X] of the powers of the prime unitary ideals of Int(Z) (Lemma 3.1). In Remark 2 we give a description of the structure of the set of these contractions; that allows us to give the primary decomposition of the ideal $I_{p^{n}}=p^{n}\mathrm{Int}(Z)\cap Z[X]$, made up of those polynomials whose fixed divisor is divisible by a prime power $p^{n}$. We shall see that we have to distinguish two cases: p⩽n and p>n (see also the examples in Remark 3). In Theorem 3.1 we describe $I_{p^{n}}$ in the case p⩽n. This result was already known in a slightly different context by Dickson (see [7, p. 22, Theorem 27]), but our different proof uses the primary decomposition of $I_{p^{n}}$ and that gives an insight to generalize the result to the second case. In Proposition 3.2 we give a set of generators for the primary components of $I_{p^{n}}$, in the case p>n. Finally in the last section, as an application, we explicitly compute the ideal $I_{p^{p+1}}$.

## 2 Fixed divisor via Spec(Int(Z))

The study of the prime spectrum of the ring Int(Z) began in [3]. We recall that the prime ideals of Int(Z) are divided into two different categories, unitary and non-unitary. Let *P* be a prime ideal of Int(Z). If it is unitary then its intersection with the ring of integers is a principal ideal generated by a prime *p*.

**Non-unitary prime ideals:**P∩Z={0}.

In this case *P* is a prime (non-maximal) ideal and it is of the form

$$B_{q}=qQ[X]\cap \mathrm{Int}(Z)$$

for some q∈Q[X] irreducible. By Gauss Lemma we may suppose that q∈Z[X] is irreducible and primitive.

**Unitary prime ideals:**P∩Z=pZ.

In this case *P* is maximal and is of the form

$$M_{p,\alpha }=\left\{f\in \mathrm{Int}(Z)|f(\alpha )\in pZ_{p}\right\}$$

for some *p* prime in Z and some $\alpha \in Z_{p}$, the ring of *p*-adic integers. We have $M_{p,\alpha }=M_{q,\beta }$ if and only if (p,α)=(q,β). So if we fix the prime *p*, the elements of $Z_{p}$ are in bijection with the unitary prime ideals of Int(Z) above the prime *p*. Moreover, $M_{p,\alpha }$ is height 1 if and only if *α* is transcendental over Q. If *α* is algebraic over Q and q(X) is its minimal polynomial then $M_{p,\alpha }⊃B_{q}$. We have $B_{q}\subset M_{p,\alpha }$ if and only if q(α)=0. Every prime ideal of Int(Z) is not finitely generated.

For a detailed study of Spec(Int(Z)) see [4].

If we denote by $d(f,Z_{p})$ the fixed divisor of f∈Int(Z) viewed as a polynomial over the ring of *p*-adic integers $Z_{p}$ (that is, $d(f,Z_{p})$ is the ideal $\left(f(\alpha )|\alpha \in Z_{p}\right)$), Gunji and McQuillan in [8] observed that

$$d(f)=\underset{p}{⋂}d(f,Z_{p})$$

where the intersection is taken over the set of primes in Z. Moreover, $d(f,Z_{p})=d(f)Z_{p}\subset Z_{p}$. Remember that given an ideal I⊂Z and a prime *p* we have $IZ_{p}=Z_{p}$ if and only if I⊄(p), so that in the previous equation we have a finite intersection. Since $Z_{p}$ is a DVR we have $d(f,Z_{p})=p^{n}Z_{p}$, for some integer *n* (which of course depends on *p*), so that the exact power of *p* which divides f(Z) is the same as the power of *p* dividing $f(Z_{p})$. Without loss of generality, we can restrict our attention to the *p*-part of the fixed divisor of a polynomial f∈Z[X]. We begin our research by finding those polynomials in Z[X] whose fixed divisor is divisible by a fixed prime *p*, namely the ideal pInt(Z)∩Z[X].

Lemma 2.1 *Let p be a prime and* $\alpha \in Z_{p}$ *. Then* $M_{p,\alpha }\cap Z[X]=(p,X-a)$ *, where* a∈Z *is such that* α≡a(modp) *. Moreover, if* $\beta \in Z_{p}$ *is another p-adic integer, we have* $M_{p,\alpha }\cap Z[X]=M_{p,\beta }\cap Z[X]$ *if and only if* α≡β(modp) *.*

Proof Let *a* be an integer as in the statement of the lemma; it exists since Z is dense in $Z_{p}$ for the *p*-adic topology. We immediately see that *p* and X−a are in $M_{p,\alpha }$. Then the conclusion follows since (p,X−a) is a maximal ideal of Z[X] and $M_{p,\alpha }\cap Z[X]$ is not equal to the whole ring Z[X]. The second statement follows from the fact that (p,X−a)=(p,X−b) if and only if a≡b(modp). □

We have just seen that the contraction of $M_{p,\alpha }$ to Z[X] depends only on the residue class modulo *p* of *α*. So, if *p* is a fixed prime, the contractions of $M_{p,\alpha }$ to Z[X] as *α* ranges through $Z_{p}$ are made up of *p* distinct maximal ideals, namely

$$\left\{M_{p,\alpha }\cap Z[X]|\alpha \in Z_{p}\right\}=\left\{\left(p,X-j)\right|j\in \{0,\ldots ,p-1\}\right\}.$$

Conversely, the set of prime ideals of Int(Z) above a fixed maximal ideal of the form (p,X−j) is $\left\{\;M_{p,\alpha }|\alpha \in Z_{p},\;\alpha \equiv j\;(\mathrm{mod}\;p)\right\}$, since $B_{q}$ are non-unitary ideals and *p* is the only prime integer in $M_{p,\alpha }$.

For a prime *p* and an integer j∈{0,…,p−1}, we set:

$$M_{p,j}=M_{j}≑(p,X-j).$$

Whenever the notation $M_{p,j}$ is used, it will be implicit that j∈{0,…,p−1}.

The next lemma computes the intersection of the ideals $M_{p,j}$, for a fixed prime *p*, by finding an ideal whose primary decomposition is given by this intersection (and its primary components are precisely the *p* ideals $M_{p,j}$). From now on we will omit the index *p*.

Lemma 2.2 *Let*p∈Z*be a prime. Then we have*

$$\underset{j=0,\ldots ,p-1}{⋂}M_{j}=\left(p,\prod_{j=0,\ldots ,p-1}(X-j)\right).$$

Proof Let *J* be the ideal on the right-hand side. If *P* is a prime minimal over *J*, then we see immediately that $P=M_{j}$ for some j∈{0,…,p−1}, since $M_{j}$ is a maximal ideal. Conversely, every such a maximal ideal contains *J* and is minimal over it. Then the minimal primary decomposition of *J* is of the form

$$J=\underset{j=0,\ldots ,p-1}{⋂}Q_{j}$$

where $Q_{j}$ is an $M_{j}$-primary ideal. Since $X-i\notin M_{j}$ for all i∈{0,…,p−1}∖{j}, we have $(X-j)\in Q_{j}$, so indeed $Q_{j}=(p,X-j)$ for each j=0,…,p−1.  □

The next proposition characterizes the principal unitary ideals in Int(Z) generated by a prime *p*.

Proposition 2.1 *Let*p∈Z*be a prime. Then the principal unitary ideal*pInt(Z)*is equal to*

$$p\mathrm{Int}(Z)=\underset{\alpha \in Z_{p}}{⋂}M_{p,\alpha }.$$

Proof We trivially have that pInt(Z) is contained in the above intersection, since *p* is in every ideal of the form $M_{p,\alpha }$. On the other hand, this intersection is equal to $\left\{f\in \mathrm{Int}(Z)|f(Z_{p})\subset pZ_{p}\right\}$. If f(X) is in this intersection, since f(X) is integer-valued and $pZ_{p}\cap Z=pZ$, we have f(Z)⊂pZ. This is equivalent to saying that f(X)/p∈Int(Z), that is, f∈pInt(Z).  □

In particular, the previous proposition implies that Int(Z) does not have the finite character property (we recall that a ring has this property if every non-zero element is contained in a finite number of maximal ideals).

From the above results we get the following theorem, which characterizes the ideal of polynomials with integer coefficients whose fixed divisor is divisible by a prime *p*, that is, the ideal pInt(Z)∩Z[X].

Theorem 2.1 *Let p be a prime. Then*

$$p\mathrm{Int}(Z)\cap Z[X]=\left(p,\prod_{j=0,\ldots ,p-1}(X-j)\right).$$

Notice that Lemma 2.2 gives the primary decomposition of pInt(Z)∩Z[X], so $M_{j}$ for j=0,…,p−1 are exactly the prime ideals belonging to it. As a consequence of this theorem we get the following well-known result: if f∈Z[X] is primitive and *p* is a prime such that d(f)⊆p then p⩽deg(f). This immediately follows from the theorem, since the degree of $\prod_{j=0,\ldots ,p-1}(X-j)$ is *p*.

We remark that by Fermatʼs little theorem the ideal on the right-hand side of the statement of Theorem 2.1 is equal to $\left(p,X^{p}-X\right)$. This amounts to saying that the two polynomials X⋅⋯⋅(X−(p−1)) and $X^{p}-X$ induce the same polynomial function on Z/pZ.

## 3 Contraction of primary ideals

We remark that Proposition 2.1 also follows from a general result contained in [11]: every unitary ideal in Int(Z) is an intersection of powers of unitary prime ideals (namely the maximal ideals $M_{p,\alpha }$). In particular, every $M_{p,\alpha }$-primary ideal is a power of $M_{p,\alpha }$ itself, since $M_{p,\alpha }$ is maximal. From the same result we also have the following characterization of the powers of $M_{p,\alpha }$, for any positive integer *n*:

$$M_{p,\alpha }^{n}=\left\{f\in \mathrm{Int}(Z)|f(\alpha )\in p^{n}Z_{p}\right\}.$$

This fact implies the following expression for the principal unitary ideal generated by $p^{n}$:

(1) $$p^{n}\mathrm{Int}(Z)=\underset{\alpha \in Z_{p}}{⋂}M_{p,\alpha }^{n}.$$

We remark again that the previous ideal is made up of those integer-valued polynomials whose extended fixed divisor is contained in $p^{n}\mathrm{Int}(Z)$. Similarly to the previous case n=1 (see Theorem 2.1) we want to find the contraction of this ideal to Z[X], in order to find the polynomials in Z[X] whose fixed divisor is divisible by $p^{n}$. We set:

(2) $$I_{p^{n}}≑p^{n}\mathrm{Int}(Z)\cap Z[X].$$

Notice that by (1) we have $I_{p^{n}}={⋂}_{\alpha \in Z_{p}}(M_{p,\alpha }^{n}\cap Z[X])$.

Like before, we begin by finding the contraction to Z[X] of $M_{p,\alpha }^{n}$, for each $\alpha \in Z_{p}$. The next lemma is a generalization of Lemma 2.1.

Lemma 3.1 *Let p be a prime, n a positive integer and* $\alpha \in Z_{p}$ *. Then* $M_{p,\alpha }^{n}\cap Z[X]=(p^{n},X-a)$ *, where* a∈Z *is such that* $\alpha \equiv a\;(\mathrm{mod}\;p^{n})$ *. The ideal* $M_{p,\alpha }^{n}\cap Z[X]$ *is* $M_{p,j}$ *-primary, where* j≡α(modp) *. Moreover, if* $\beta \in Z_{p}$ *is another p-adic integer, we have* $M_{p,\alpha }^{n}\cap Z[X]=M_{p,\beta }^{n}\cap Z[X]$ *if and only if* $\alpha \equiv \beta \;(\mathrm{mod}\;p^{n})$ *.*

Proof The case n=1 has been done in Lemma 2.1. For the general case, let a∈Z be such that $a\equiv \alpha \;(\mathrm{mod}\;p^{n})$ (again, such an integer exists since Z is dense in $Z_{p}$ for the *p*-adic topology). We have $\left(p^{n},X-a)\subset M_{p,\alpha }^{n}\cap Z[X\right]$ (notice that if n>1 then $\left(p^{n},X-a\right)$ is not a prime ideal). To prove the other inclusion let $f\in M_{p,\alpha }^{n}\cap Z[X]$. By the Euclidean algorithm in Z[X] (the leading coefficient of X−a is a unit) we have

f(X)=q(X)(X−a)+f(a).

Since $f(\alpha )\in p^{n}Z_{p}$ and $p^{n}|a-\alpha$ we have $p^{n}|f(a)$. Hence, $f\in (p^{n},X-a)$ as we wanted. Since $M_{p,\alpha }^{n}$ is an $M_{p,\alpha }$-primary ideal in Int(Z) and the contraction of a primary ideal is a primary ideal, by Lemma 2.1 we get the second statement. Finally, like in the proof of Lemma 2.1, we immediately see that $\left(p^{n},X-a)=(p^{n},X-b\right)$ if and only if $a\equiv b\;(\mathrm{mod}\;p^{n})$, which gives the last statement of the lemma. □

Remark 1 It is worth to write down the fact that we used in the above proof: given a polynomial f∈Z[X], we have

(3) $$f\in \left(p^{n},X-a\right)\;⟺\;f(a)\equiv 0\;\left(\mathrm{mod}\;p^{n}\right).$$

Remark 2 If *p* is a fixed prime and *n* is a positive integer, Lemma 3.1 implies

$$I_{p,n}≑\left\{M_{p,\alpha }^{n}\cap Z[X]|\alpha \in Z_{p}\right\}=\left\{\left(p^{n},X-i\right)|i=0,\ldots ,p^{n}-1\right\}.$$

Let us consider an ideal $I=M_{p,\alpha }^{n}\cap Z[X]=(p^{n},X-i)$ in $I_{p,n}$, with i∈Z, $i\equiv \alpha \;(\mathrm{mod}\;p^{n})$. It is quite easy to see that *I* contains ${\left(M_{p,\alpha }\cap Z[X]\right)}^{n}=M_{p,j}^{n}={\left(p,X-j\right)}^{n}$, where j∈{0,…,p−1}, j≡α(modp) (notice that j≡i(modp)). If n>1 this containment is strict, since $X-i\notin {\left(p,X-j\right)}^{n}$. We can group the ideals of $I_{p,n}$ according to their radical: there are *p* radicals of these $p^{n}$ ideals, namely the maximal ideals $M_{p,j}$, j=0,…,p−1. This amounts to making a partition of the residue classes modulo $p^{n}$ into *p* different sets of elements congruent to *j* modulo *p*, for j=0,…,p−1; each of these sets has cardinality $p^{n-1}$. Correspondingly we have:

$$I_{p,n}=\underset{j=0,\ldots ,p-1}{⋃}I_{p,n,j}$$

where $I_{p,n,j}≑\{(p^{n},X-i)|i=0,\ldots ,p^{n}-1,\;i\equiv j\;(\mathrm{mod}\;p)\}$, for j=0,…,p−1. Every ideal in $I_{p,n,j}$ is $M_{p,j}$-primary and it contains the *n*-th power of its radical, namely $M_{p,j}^{n}$.

Now we want to compute the intersection of the ideals in $I_{p,n}$, which is equal to the ideal $I_{p^{n}}$ in Z[X] (see (1) and (2)). We can express this intersection as an intersection of $M_{p,j}$-primary ideals as we have said above, in the following way (in the first equality we make use of Eq. (1) and Lemma 3.1):

(4) $$I_{p^{n}}=\underset{i=0,\ldots ,p^{n}-1}{⋂}\left(p^{n},X-i\right)=\underset{j=0,\ldots ,p-1}{⋂}Q_{p,n,j}$$

where

$$Q_{p,n,j}≑\underset{i\equiv j\;(\mathrm{mod}\;p)}{⋂}\left(p^{n},X-i\right)$$

(notice that the intersection is taken over the set $\left\{i\in \{0,\ldots ,p^{n}-1\}|i\equiv j\;(\mathrm{mod}\;p)\right\}$). The ideal $Q_{p,n,j}$ is an $M_{p,j}$-primary ideal, for j=0,…,p−1, since the intersection of *M*-primary ideals is an *M*-primary ideal. We will omit the index *p* in $Q_{p,n,j}$ and in $M_{p,j}$ if that will be clear from the context. The $M_{p,j}$-primary ideal $Q_{n,j}$ is just the intersection of the ideals in $I_{p,n,j}$, according to the partition we made. It is equal to the set of polynomials in Z[X] which modulo $p^{n}$ are zero at the residue classes congruent to *j* modulo *p* (see (3) of Remark 1). We remark that (4) is the minimal primary decomposition of $I_{p^{n}}$. Notice that there are no embedded components in this primary decomposition, since the prime ideals belonging to it (the minimal primes containing $I_{p^{n}}$) are $\left\{M_{j}|j=0,\ldots ,p-1\right\}$, which are maximal ideals.

We recall that if *I* and *J* are two coprime ideals in a ring *R*, that is I+J=R, then IJ=I∩J (in general only the inclusion IJ⊂I∩J holds). The condition for two ideals *I* and *J* to be coprime amounts to saying that *I* and *J* are not contained in a same maximal ideal *M*, that is, I+J is not contained in any maximal ideal *M*. If $M_{1}$ and $M_{2}$ are two distinct maximal ideals then they are coprime, and the same holds for any of their respective powers. If *R* is Noetherian, then every primary ideal *Q* contains a power of its radical and moreover if the radical of *Q* is maximal then also the converse holds (see [14]). So if $Q_{i}$ is an $M_{i}$-primary ideal for i=1,2 and $M_{1},M_{2}$ are distinct maximal ideals, then $Q_{1}$ and $Q_{2}$ are coprime.

Since ${\left\{M_{j}\right\}}_{j=0,\ldots ,p-1}$ are *p* distinct maximal ideals, for what we have just said above we have

$$\underset{j=0,\ldots ,p-1}{⋂}Q_{n,j}=\prod_{j=0,\ldots ,p-1}Q_{n,j}.$$

Now we want to describe the $M_{j}$-primary ideals $Q_{n,j}$, for j=0,…,p−1. The next lemma gives a relation of containment between these ideals and the *n*-th powers of their radicals.

Lemma 3.2 *Let p be a fixed prime and n a positive integer. For each* j=0,…,p−1 *, we have*

$$Q_{n,j}⊇M_{j}^{n}.$$

Proof The statement follows from Remark 2. □

As a consequence of this lemma, we get the following result:

Corollary 3.1 *Let p be a fixed prime and n a positive integer. Then we have*:

$$I_{p^{n}}⊇{\left(p,\prod_{j=0,\ldots ,p-1}(X-j)\right)}^{n}.$$

Proof By (4) and Lemma 3.2 we have

$$I_{p^{n}}=\prod_{j=0,\ldots ,p-1}Q_{n,j}⊇\prod_{j=0,\ldots ,p-1}M_{j}^{n}$$

where the last containment follows from Lemma 3.2. Finally, by Lemma 2.2, the product of the ideals $M_{j}^{n}$ is equal to

$$\prod_{j=0,\ldots ,p-1}M_{j}^{n}={\left(p,\prod_{j=0,\ldots ,p-1}(X-j)\right)}^{n}.$$

Notice that the product of the $M_{j}$ʼs is actually equal to their intersection, since they are maximal coprime ideals. □

The last formula of the previous proof gives the primary decomposition of the ideal ${\left(p,\prod_{j=0,\ldots ,p-1}(X-j)\right)}^{n}$.

Remark 3 In general, for a fixed j∈{0,…,p−1}, the reverse containment of Lemma 3.2 does not hold, that is, the *n*-th power of $M_{j}$ can be strictly contained in the $M_{j}$-primary ideal $Q_{n,j}$. For example (again, we use (3) to prove the containment):

$$X(X-2)\in \left(\underset{k=0,\ldots ,3}{⋂}\left(2^{3},X-2k\right)\right)\setminus {\left(2,X\right)}^{3}.$$

Because of that, in general we do not have an equality in Corollary 3.1. For example, let p=2 and n=3. We have

$$X(X-1)(X-2)(X-3)\in I_{2^{3}}\setminus {\left(2,X(X-1)\right)}^{3}.$$

It is also false that

$$\underset{i=0,\ldots ,p^{n}-1}{⋂}\left(p^{n},X-i\right)=\left(p^{n},\prod_{i=0,\ldots ,p^{n}-1}(X-i)\right).$$

See for example: p=2, n=2: $2X(X-1)\in {⋂}_{i=0,\ldots ,3}(4,X-i)\setminus (4,\prod_{i=0,\ldots ,3}(X-i))$.

We want to study under which conditions the ideal $Q_{n,j}$ is equal to $M_{j}^{n}$. Our aim is to find a set of generators for $Q_{n,j}$. For $f\in Q_{n,j}$, we have $f\in (p^{n},X-i)$ for each i≡j(modp), $i\in \{0,\ldots ,p^{n}-1\}$. By (3) that means $p^{n}|f(i)$ for each such an *i*. Equivalently, such a polynomial has the property that modulo $p^{n}$ it is zero at the $p^{n-1}$ residue classes of $Z/p^{n}Z$ which are congruent to *j* modulo *p*.

Without loss of generality, we proceed by considering the case j=0. We set $M=M_{0}=(p,X)$ and $Q_{n}=Q_{n,0}={⋂}_{i\equiv 0\;(\mathrm{mod}\;p)}(p^{n},X-i)$. Let $f\in Q_{n}$, of degree *m*. We have

(5) $$f(X)=q_{1}(X)X+f(0)$$

where $q_{1}\in Z[X]$ has degree equal to m−1. Since $f\in (p^{n},X)$ we have $p^{n}|f(0)$.

Since $f\in (p^{n},X-p)$, we have $p^{n}|f(p)=q_{1}(p)p+f(0)$, so $p^{n-1}|q_{1}(p)$. By the Euclidean algorithm,

(6) $$q_{1}(X)=q_{2}(X)(X-p)+q_{1}(p)$$

for some polynomial $q_{2}\in Z[X]$ of degree m−2. So

$$f(X)=q_{2}(X)(X-p)X+q_{1}(p)X+f(0).$$

We set $R_{1}(X)=q_{1}(p)X+f(0)$. Then $R_{1}\in M^{n}$, since $p^{n-1}|q_{1}(p)$ and $p^{n}|f(0)$. Since $f\in (p^{n},X-2p)$, we have $p^{n}|f(2p)=q_{2}(2p)2p^{2}+q_{1}(p)2p+f(0)$. If p>2 then $p^{n-2}|q_{2}(2p)$, because $p^{n}|q_{1}(p)2p+f(0)$. If p=2 then we can just say $p^{n-3}|q_{2}(2p)$. By the Euclidean algorithm again, we have

$$q_{2}(X)=q_{3}(X)(X-2p)+q_{2}(2p)$$

for some $q_{3}\in Z[X]$. So we have

$$f(X)=q_{3}(X)(X-2p)(X-p)X+q_{2}(2p)(X-p)X+q_{1}(p)X+f(0).$$

Like before, if we set $R_{2}(X)=q_{2}(2p)(X-p)X+q_{1}(p)X+f(0)$, we have $R_{2}\in M^{n}$ if p>2, or $R_{2}\in Q_{n}$ if p=2.

We define now the following family of polynomials:

Definition 3.1 For each k∈N, k⩾1, we set

$$G_{p,0,k}(X)=G_{k}(X)≑\prod_{h=0,\ldots ,k-1}(X-hp).$$

We also set $G_{0}(X)≑1$.

From now on, we will omit the index *p* in the above notation.

Notice that the polynomials $G_{k}(X)$, whose degree for each *k* is *k*, enjoy these properties:

i) For every t∈Z, $G_{k}(tp)=p^{k}t(t-1)\cdots (t-(k-1))$. Hence, the highest power of *p* which divides all the integers in the set $\left\{G_{k}(tp)|t\in Z\right\}$ is $p^{k+v_{p}(k!)}$. It is easy to see that $k+v_{p}(k!)=v_{p}((pk)!)$.
ii) $G_{k}(X)=(X-kp)G_{k-1}(X)$.
iii) since for every integer *h*, X−hp∈M, we have $G_{k}(X)\in M^{k}$. We remark that *k* is the maximal integer with this property, since $\deg(G_{k})=k$ and $G_{k}(X)$ is primitive (since monic).

Recall that, by Lemma 3.2, for every integer *n* we have $Q_{n}⊇M^{n}$. By property iii) above $G_{k}\in M^{n}$ if and only if n⩽k. By property i) we have $G_{k}\in Q_{n}$ if and only if $k+v_{p}(k!)⩾n$. From these remarks, it is very easy to deduce that, in the case p⩾n, if $G_{k}\in Q_{n}$ then $G_{k}\in M^{n}$. In fact, if that is not the case, it follows from above that k<n. Since n⩽p we get $k+v_{p}(k!)=k$. Since $G_{k}\in Q_{n}$, we have n⩽k, contradiction.

The next lemma gives a sort of division algorithm between an element of $Q_{n}$ and the polynomials ${\left\{G_{k}(X)\right\}}_{k\in N}$. In particular, we will deduce that $Q_{n}=M^{n}$, if p⩾n.

Lemma 3.3 *Let p be a prime and n a positive integer. Let* $f\in Q_{p,n,0}=Q_{n}$ *be of degree m. Then for each* 1⩽k⩽m *there exists* $q_{k}\in Z[X]$ *of degree* m−k *such that*

$$f(X)=q_{k}(X)G_{k}(X)+R_{k-1}(X)$$

*where* $R_{k-1}(X)≑\sum_{h=1,\ldots ,k-1}q_{h}(hp)G_{h}(X)$ *for* k⩾2 *and* $R_{0}(X)≑f(0)$ *.*

*We also have*$q_{k}(X)=q_{k+1}(X)(X-kp)+q_{k}(kp)$*for*k=1,…,m−1*. Moreover, for each such a k the following hold*:

i) $p^{n-v_{p}((pk)!)}|q_{k}(kp)$*, if*$v_{p}((pk)!)<n$*.*
ii) $q_{k}(kp)G_{k}(X)\in Q_{n}$*and if*k<p*then*$q_{k}(kp)G_{k}(X)\in M^{n}$*.*
iii) *If*m⩽p*then*$R_{k-1}\in M^{n}$*for*k=1,…,m*.*
  *If*m>p*then*$R_{k-1}\in M^{n}$*for*k=1,…,p*and*$R_{k-1}\in Q_{n}$*for*k=p+1,…,m*.*

Proof We proceed by induction on *k*. The case k=1 follows from (5), and by (6) we have the last statement regarding the relation between $q_{1}(X)$ and $q_{2}(X)$. Suppose now the statement is true for k−1, so that

$$f(X)=q_{k-1}(X)G_{k-1}(X)+R_{k-2}(X)$$

with $R_{k-2}(X)≑\sum_{h=1,\ldots ,k-2}q_{h}(hp)G_{h}(X)$ and

– $p^{n-v_{p}((p(k-1))!)}|q_{k-1}((k-1)p)$, if $v_{p}((p(k-1)!))<n$,
– $q_{k-1}((k-1)p)G_{k-1}(X)$ belongs to $Q_{n}$ and if k−1<p it belongs to $M^{n}$,
– $R_{k-2}\in Q_{n}$ and if k−2<p then $R_{k-2}\in M^{n}$.

We divide $q_{k-1}(X)$ by (X−(k−1)p) and we get

$$q_{k-1}(X)=q_{k}(X)\left(X-(k-1)p\right)+q_{k-1}\left((k-1)p\right)$$

for some polynomial $q_{k}\in Z[X]$ of degree m−k. We substitute this expression of $q_{k-1}(X)$ in the equation of f(X) at the step k−1 and we get:

(7) $$f(X)=q_{k}(X)\left(X-(k-1)p\right)G_{k-1}(X)+R_{k-1}(X),$$

where $R_{k-1}(X)≑q_{k-1}((k-1)p)G_{k-1}(X)+R_{k-2}(X)$. This is the expression of f(X) at step *k*, since $\left(X-(k-1)p)G_{k-1}(X\right)$ is equal to $G_{k}(X)$. By the inductive assumption, $R_{k-1}\in Q_{n}$ and if k−1<p we also have $R_{k-1}\in M^{n}$. We still have to verify i) and ii).

We evaluate the expression (7) in X=kp and we get

$$f(kp)=q_{k}(kp)G_{k}(kp)+R_{k-1}(kp)=q_{k}(kp)p^{k}k!+R_{k-1}(kp).$$

Since $p^{n}$ divides both f(kp) and $R_{k-1}(kp)$ (by definition of $Q_{n}$), if $v_{p}((pk)!)<n$ we get that $q_{k}(kp)$ is divisible by $p^{n-v_{p}((pk)!)}$, which is statement i) at the step *k*. Notice that $q_{k}(kp)G_{k}(X)$ is zero modulo $p^{n}$ on every integer congruent to zero modulo *p*; hence, $q_{k}(kp)G_{k}(X)\in Q_{n}$. Moreover, $k<p\Leftrightarrow v_{p}(k!)=0$, so in that case $q_{k}(kp)G_{k}(X)\in M^{n}$. So ii) follows. □

Notice that by formula (3) of Remark 1, under the assumptions of Lemma 3.3 we have for each k∈{1,…,p−1} that

$$q_{k}\in \left(p^{n-k},X-kp\right)$$

(see i) of Lemma 3.3: in this case $v_{p}((pk)!)=k$). If k=m=deg(f) then $q_{k}\in Z$. Hence, we get the following expression for a polynomial $f\in Q_{n}$ in the case p⩾n>m (this assumption is not restrictive, since $X^{n}\in Q_{n}$):

(8) $$f(X)=q_{m}G_{m}(X)+R_{m-1}(X)=q_{m}G_{m}(X)+\sum_{k=1,\ldots ,m-1}q_{k}(kp)G_{k}(X)$$

where $q_{m}\in Z$ is divisible by $p^{n-m}$ and $R_{m-1}(X)$ is in $M^{n}$.

The next proposition determines the primary components $Q_{n,j}$ of $I_{p^{n}}$ of (4) in the case p⩾n. It shows that in this case the containment of Lemma 3.2 is indeed an equality.

Proposition 3.1 *Let* p∈Z *be a prime and n a positive integer such that* p⩾n *. Then for each* j=0,…,p−1 *we have*

$$Q_{n,j}=M_{j}^{n}.$$

Proof It is sufficient to prove the statement for j=0: for the other cases we consider the Z[X]-automorphisms $\pi_{j}(X)=X-j$, for j=1,…,p−1, which permute the ideals $Q_{n,j}$ and $M_{j}$. Let $Q_{n}=Q_{n,0}$ and $M=M_{0}$.

The inclusion (⊇) follows from Lemma 3.2. For the other inclusion (⊆), let f(X) be in $Q_{n}$. We can assume that the degree *m* of f(X) is less than *n*, since $X^{n}$ is the smallest monic monomial in $Q_{n}$. By Eq. (8) above, f(X) is in $M^{n}$, since $p^{n-m}$ divides $q_{m}$, $G_{m}\in M^{m}$ and $R_{m-1}\in M^{n}$ by Lemma 3.3 (notice that m−1<p). □

Remark 4 In the case p⩾n, Lemma 3.3 implies that $Q_{n}$ is generated by ${\left\{p^{n-m}G_{m}(X)\right\}}_{0⩽m⩽n}$: it is easy to verify that these polynomials are in $Q_{n}$ (using (3) again) and (8) implies that every polynomial $f\in Q_{n}$ is a Z-linear combination of ${\left\{p^{n-m}G_{m}(X)\right\}}_{0⩽m⩽n}$, since $q_{m}(mp)$ is divisible by $p^{n-m}$, for each of the relevant *m*.

The following theorem gives a description of the ideal $I_{p^{n}}$ in the case p⩾n. In this case the containment of Corollary 3.1 becomes an equality.

Theorem 3.1 *Let* p∈Z *be a prime and n a positive integer such that* p⩾n *. Then the ideal in* Z[X] *of those polynomials whose fixed divisor is divisible by* $p^{n}$ *is equal to*

$$I_{p^{n}}={\left(p,\prod_{i=0,\ldots ,p-1}(X-i)\right)}^{n}.$$

Proof By Proposition 3.1, for each j=0,…,p−1 the ideal $Q_{n,j}$ is equal to $M_{j}^{n}$. So, by the last formula of the proof of Corollary 3.1, we get the statement. □

As a consequence, we have the following remark. Let *p* be a prime and *n* a positive integer less than or equal to *p*. Let $f\in I_{p^{n}}$ such that the content of f(X) is not divisible by *p*. Then deg(f)⩾np, since $np=\deg(\prod_{i=0,\ldots ,p-1}{\left(X-i\right)}^{n})$. Another well-known result in this context is the following: if we fix the degree *d* of such a polynomial *f*, then the maximum *n* such that $f\in I_{p^{n}}$ is bounded by $n⩽\sum_{k⩾1}[d/p^{k}]=v_{p}(d!)$.

If we drop the assumption p⩾n, the ideal $Q_{n,j}$ may strictly contain $M_{j}^{n}$, as we observed in Remark 3. The next proposition shows that this is always the case, if p<n. This result follows from Lemma 3.3 as Proposition 3.1 does, and it covers the remaining case p<n. It is stated for the case j=0. Remember that M=(p,X) and $Q_{n}={⋂}_{i\equiv 0\;(\mathrm{mod}\;p)}(p^{n},X-i)$.

Proposition 3.2 *Let*p∈Z*be a prime and n a positive integer such that*p<n*. Then we have*

$$Q_{n}=M^{n}+\left(q_{n,p}G_{p}(X),\ldots ,q_{n,n-1}G_{n-1}(X)\right)$$

*where, for each*k=p,…,n−1*,*$q_{n,k}$*is an integer defined as follows*:

$$q_{n,k}≑\left\{ \begin{array}{cc} p^{n-v_{p}((pk)!)}, & \text{if}v_{p}((pk)!)<n,\\ 1, & \text{otherwise.} \end{array}\right.$$

*In particular,*$M^{n}$*is strictly contained in*$Q_{n}$*.*

Proof We begin by proving the containment (⊇). Lemma 3.2 gives $M^{n}\subseteq Q_{n}$. We have to show that the polynomials $q_{n,k}G_{k}(X)$, for k∈{p,…,n−1}, lie in $Q_{n}$. This follows from property i) of the polynomials $G_{k}(X)$ and the definition of $q_{n,m}$.

Now we prove the other containment (⊆). Let $f\in Q_{n}$ be of degree *m*. If m<p then $f\in M^{n}$ (see Lemma 3.3 and in particular (8)). So we suppose p⩽m. By Lemma 3.3 we have

(9) $$f(X)=\sum_{k=p,\ldots ,m}q_{h}(hp)G_{h}(X)+R_{p-1}(X)$$

where $R_{p-1}(X)=\sum_{k=1,\ldots ,p-1}q_{k}(hp)G_{h}(X)\in M^{n}$ and $q_{m}\in Z$, so that $q_{m}(mp)=q_{n,m}$. Then, since $q_{n,k}=p^{n-v_{p}((pk)!)}|q_{k}(kp)$ if $v_{p}((pk)!)<n$, it follows that the first sum on the right-hand side of the previous equation belongs to the ideal $\left(q_{n,p}G_{p}(X),\ldots ,q_{n,n-1}G_{n-1}(X)\right)$. For the last sentence of the proposition, we remark that the polynomials ${\left\{q_{n,k}G_{k}(X)\right\}}_{k=p,\ldots ,n-1}$ are not contained in $M^{n}$: in fact, for each k∈{p,…,n−1}, by property iii) of the polynomials $G_{k}(X)$ we have that the minimal integer *N* such that $q_{n,k}G_{k}(X)$ is contained in $M^{N}$ is $n-v_{p}(k!)$ if $v_{p}((pk)!)=k+v_{p}(k!)<n$ and it is *k* otherwise. In both cases it is strictly less than *n* (since $v_{p}(k!)⩾1$, if k⩾p). □

Remark 5 The following remark allows us to obtain another set of generators for $Q_{n}$. We set

(10) $$\bar{m}=\bar{m}(n,p)≑\min\left\{m\in N|v_{p}\left((pm)!\right)⩾n\right\}.$$

Remember that $v_{p}((pm)!)=m+v_{p}(m!)$. If p⩾n then $\bar{m}=n$ and if p<n then $p⩽\bar{m}<n$.

Suppose p<n. Then for each $m\in \{\bar{m},\ldots ,n\}$ we have $v_{p}((pm)!)⩾n$, since the function $e(m)=m+v_{p}(m!)$ is increasing. So for each such *m* we have $q_{n,m}=1$, hence $G_{m}\in (G_{\bar{m}}(X))$. So we have the equalities:

(11) $$Q_{n}=M^{n}+\left(q_{n,m}G_{m}(X)|m=p,\ldots ,\bar{m}\right)=\left(q_{n,m}G_{m}(X)|m=0,\ldots ,\bar{m}\right)$$

where $q_{n,m}=p^{n-m}$, for m=0,…,p−1, and for $m=p,\ldots ,\bar{m}$ is defined as in the statement of Proposition 3.2. The containment (⊇) is just an easy verification using the properties of the polynomials $G_{m}(X)$; the other containment follows by (9).

We can now group together Proposition 3.1, Proposition 3.2 into the following one:

Proposition 3.3 *Let*p∈Z*be a prime and n a positive integer. Then we have*

$$Q_{n}=\left(q_{n,0}G_{0}(X),\ldots ,q_{n,\bar{m}}G_{\bar{m}}(X)\right)$$

*where*$\bar{m}=\min\{m\in N|v_{p}((pm)!)⩾n\}$*and for each*$m=0,\ldots ,\bar{m}$*,*$q_{n,m}$*is an integer defined as follows*:

$$q_{n,m}≑\left\{ \begin{array}{cc} p^{n-v_{p}((pm)!)}, & m<\bar{m},\\ 1, & m=\bar{m}. \end{array}\right.$$

It is clear what the primary ideals $Q_{j}$, for j=1,…,p−1, look like:

$$Q_{n,j}=\underset{i\equiv j\;(\mathrm{mod}\;p)}{⋂}\left(p^{n},X-i\right)=M_{j}^{n}+\left(q_{n,p}G_{p}(X-j),\ldots ,q_{n,\bar{m}}G_{\bar{m}}(X-j)\right)=\left(q_{n,0}G_{0}(X-j),\ldots ,q_{n,\bar{m}}G_{\bar{m}}(X-j)\right).$$

In fact, for each j=1,…,p−1, it is sufficient to consider the automorphisms of Z[X] given by $\pi_{j}(X)=X-j$. It is straightforward to check that $\pi_{j}(I_{p^{n}})=I_{p^{n}}$. Moreover, $\pi (Q_{n,0})=Q_{n,j}$ and $\pi (M_{0})=M_{j}$ for each such a *j*, so that $\pi_{j}$ permutes the primary components of the ideal $I_{p^{n}}$.

The ideal $I_{p^{n}}=p^{n}\mathrm{Int}(Z)\cap Z[X]$ was studied in [2] in a slightly different context, as the kernel of the natural map $\varphi_{n}:Z[X]\to \Phi_{n}$, where the latter is the set of functions from $Z/p^{n}Z$ to itself. In that article a recursive formula is given for a set of generators of this ideal. Our approach gives a new point of view to describe this ideal.

For other works about the ideal $I_{p^{n}}$ in a slightly different context, see [9], [10], [13]. This ideal is important in the study of the problem of the polynomial representation of a function from $Z/p^{n}Z$ to itself.

## 4 Case $I_{p^{p+1}}$

As a corollary we give an explicit expression for the ideal $I_{p^{n}}$ in the case n=p+1. By Proposition 3.2 the primary components of $I_{p^{p+1}}$ are

(12) $$Q_{p+1,j}=M_{j}^{p+1}+\left(G_{p}(X-j)\right)$$

for j=0,…,p−1.

Corollary 4.1 $$I_{p^{p+1}}={\left(p,\prod_{i=0,\ldots ,p-1}(X-i)\right)}^{p+1}+\left(H(X)\right)$$

*where*$H(X)=\prod_{i=0,\ldots ,p^{2}-1}(X-i)$*.*

We want to stress that the polynomial H(X) is not contained in the first ideal of the right-hand side of the statement. In [2] a similar result is stated with another polynomial $H_{2}(X)$ instead of our H(X). Indeed the two polynomials, as already remarked in [2], are congruent modulo the ideal ${\left(p,\prod_{i=0,\ldots ,p-1}(X-i)\right)}^{p+1}$.

Proof of Corollary 4.1 Like before, we set $Q_{p,p+1,j}=Q_{p+1,j}$. The containment (⊇) follows from Corollary 3.1 and because the polynomial H(X) is equal to $\prod_{j=0,\ldots ,p-1}G_{p}(X-j)$ and for each j=0,…,p−1 the polynomial $G_{p}(X-j)$ is in $Q_{p+1,j}$ by Proposition 3.2. Since $Q_{p+1,j}$, for j=0,…,p−1, are exactly the primary components of $I_{p^{p+1}}$ (see (4)), we get the claim.

Now we prove the other containment (⊆). Let $f\in I_{p^{p+1}}={⋂}_{j=0,\ldots ,p-1}Q_{p+1,j}$. By (12) we have:

$$f(X)\equiv C_{p,j}(X)G_{p}(X-j)\;\left(\mathrm{mod}\;M_{j}^{p+1}\right)$$

for some $C_{p,j}\in Z[X]$, for j=0,…,p−1.

Since the ideals $\left\{M_{j}^{p+1}={\left(p,X-j\right)}^{p+1}|j=0,\ldots ,p-1\right\}$ are pairwise coprime (because they are powers of distinct maximal ideals, respectively), by the Chinese Remainder Theorem we have the following isomorphism:

(13) $$Z[X]/\left(\prod_{j=0}^{p-1}M_{j}^{p+1}\right)≅Z[X]/M_{0}^{p+1}\times \cdots \times Z[X]/M_{p-1}^{p+1}.$$

We need now the following lemma, which tells us what is the residue of the polynomial H(X) modulo each ideal $M_{j}^{p+1}$:

Lemma 4.1 *Let p be a prime and let* $H(X)=\prod_{j=0,\ldots ,p-1}G_{p}(X-j)$ *. Then for each* k=0,…,p−1 *we have*

$$H(X)\equiv -G_{p}(X-k)\;\left(\mathrm{mod}\;M_{k}^{p+1}\right).$$

Proof Let k∈{0,…,p−1} and set $I_{k}=\{0,\ldots ,p-1\}\setminus \{k\}$. For each $j\in I_{k}$ we have $G_{p}(k-j)\equiv {\left(k-j\right)}^{p}\;(\mathrm{mod}\;p)$. We have

$$H(X)+G_{p}(X-k)=G_{p}(X-k)\left[1+\prod_{j\in I_{k}}G_{p}(X-j)\right].$$

Since $G_{p}(X-k)\in M_{k}^{p}$ we have just to prove that $T_{k}(X)=1+\prod_{j\in I_{k}}G_{p}(X-j)\in M_{k}$. By formula (3) in Remark 1 it is sufficient to prove that $T_{k}(k)$ is divisible by *p*. We have

$$T_{k}(k)\equiv 1+\prod_{j\in I_{k}}{\left(k-j\right)}^{p}\;(\mathrm{mod}\;p)\equiv 1+{\left(\prod_{s=1,\ldots ,p-1}s\right)}^{p}\;(\mathrm{mod}\;p)\equiv 1+(p-1){!}^{p}\;(\mathrm{mod}\;p)\equiv {\left(1+(p-1)!\right)}^{p}\;(\mathrm{mod}\;p)$$

which is congruent to zero by Wilsonʼs theorem. □

We finish now the proof of the corollary.

By the Chinese Remainder Theorem, there exists a polynomial P∈Z[X] such that $P(X)\equiv -C_{p,j}(X)\;(\mathrm{mod}\;M_{j}^{p+1})$, for each j=0,…,p−1. Then by the previous lemma $P(X)H(X)\equiv C_{p,j}(X)G_{p}(X-j)\;(\mathrm{mod}\;M_{j}^{p+1})$ and so again by the isomorphism (13) above we have

$$f(X)\equiv P(X)H(X)\;\left(\mathrm{mod}\;\prod_{j=0,\ldots ,p-1}M_{j}^{p+1}\right)$$

so we are done since $\prod_{j=0,\ldots ,p-1}M_{j}^{p+1}={\left(p,\prod_{i=0,\ldots ,p-1}(X-i)\right)}^{p+1}$ (see the proof of Corollary 3.1). □
